# Anti-PrP^C ^monoclonal antibody infusion as a novel treatment for cognitive deficits in an alzheimer's disease model mouse

**DOI:** 10.1186/1471-2202-11-130

**Published:** 2010-10-14

**Authors:** Erika Chung, Yong Ji, Yanjie Sun, Richard J Kascsak, Regina B Kascsak, Pankaj D Mehta, Stephen M Strittmatter, Thomas Wisniewski

**Affiliations:** 1 Department of Neurology, New York University School of Medicine, 550 First Avenue, New York, NY 10016, USA; 2 New York State Institute for Basic Research in Developmental Disabilities, 1050 Forest Hill Rd., Staten Island, NY 10314, USA; 3 Cellular Neuroscience, Neurodegeneration and Repair Program, Yale University School of Medicine, 295 Congress Avenue, New Haven, CT 06536, USA; 4 Department of Pathology, New York University School of Medicine, 550 First Avenue, New York, NY 10016, USA; 5 Department of Psychiatry, New York University School of Medicine, 550 First Avenue, New York, NY 10016, USA

## Abstract

**Background:**

Alzheimer's Disease (AD) is the most common of the conformational neurodegenerative disorders characterized by the conversion of a normal biological protein into a β-sheet-rich pathological isoform. In AD the normal soluble Aβ (sAβ) forms oligomers and fibrils which assemble into neuritic plaques. The most toxic form of Aβ is thought to be oligomeric. A recent study reveals the cellular prion protein, PrP^C^, to be a receptor for Aβ oligomers. Aβ oligomers suppress LTP signal in murine hippocampal slices but activity remains when pretreated with the PrP monoclonal anti-PrP antibody, 6D11. We hypothesized that targeting of PrP^C ^to prevent Aβ oligomer-related cognitive deficits is a potentially novel therapeutic approach. APP/PS1 transgenic mice aged 8 months were intraperitoneally (i.p.) injected with 1 mg 6D11 for 5 days/week for 2 weeks. Two wild-type control groups were given either the same 6D11 injections or vehicle solution. Additional groups of APP/PS1 transgenic mice were given either i.p. injections of vehicle solution or the same dose of mouse IgG over the same period. The mice were then subjected to cognitive behavioral testing using a radial arm maze, over a period of 10 days. At the conclusion of behavioral testing, animals were sacrificed and brain tissue was analyzed biochemically or immunohistochemically for the levels of amyloid plaques, PrP^C^, synaptophysin, Aβ40/42 and Aβ oligomers.

**Results:**

Behavioral testing showed a marked decrease in errors in 6D11 treated APP/PS1 Tg mice compared with the non-6D11 treated Tg groups (p < 0.0001). 6D11 treated APP/PS1 Tg mice behaved the same as wild-type controls indicating a recovery in cognitive learning, even after this short term 6D11 treatment. Brain tissue analysis from both treated and vehicle treated APP/PS1 groups indicate no significant differences in amyloid plaque burden, Aβ40/42, PrP^C ^or Aβ oligomer levels. 6D11 treated APP/PS1 Tg mice had significantly greater synaptophysin immunoreactivity in the dentate gyrus molecular layer of the hippocampus compared to vehicle treated APP/PS1 Tg mice (p < 0.05).

**Conclusions:**

Even short term treatment with monoclonal antibodies such as 6D11 or other compounds which block the binding of Aβ oligomers to PrP^C ^can be used to treat cognitive deficits in aged AD transgenic mice.

## Background

Alzheimer's disease is the most common cause of dementia worldwide, affecting approximately 36 million people currently [1]. By 2050, according to some estimates, 1 in 85 persons worldwide will be affected by AD [1,2]. Currently available treatments for AD provide largely symptomatic relief with only minor effects on the course of the disease. The diagnostic neuropathological lesions of AD are the accumulation of Aβ as neuritic plaques and congophilic angiopathy, as well as aggregation of abnormally phosphorylated tau in the form of neurofibrillary tangles (NFTs)[3]. The dominant theory for the causation of AD has been the amyloid cascade hypothesis [4,5]. This theory currently suggests that accumulation of Aβ peptides particularly in a highly toxic oligomeric form is the primary pathogenic driver, that downstream leads to tau hyperphosphorylation, NFT formation and ultimately to synaptic and neuronal loss. A recent study using oligomers derived from synthetic Aβ peptides reported that a high affinity specific binding site for Aβ oligomers is the cellular prion protein (PrP^C^) and that PrP^C ^is a requirement for acute Aβ oligomer suppression of synaptic plasticity in hippocampal slices [6,7]. Furthermore, it was shown that a monoclonal anti-PrP antibody (mAb) 6D11 could block this Aβ oligomer mediated toxicity in hippocampal slices [6,7]. In addition it was recently shown that PrP^C ^expression is necessary for memory impairment in an AD transgenic (Tg) mouse model [8]. However, another study, while confirming that PrP^C ^is a high affinity binding site for Aβ oligomers, suggested that memory impairment induced by acute injection of Aβ oligomers derived from synthetic peptides does not require PrP^C ^[9]. We sought to test the hypothesis that short term treatment using monoclonal 6D11 could reverse memory impairment in an established APP/PS1 Tg mouse model of AD [10]. Such an approach to block *in vivo *derived Aβ oligomer mediated toxicity would represent a novel treatment strategy for AD.

## Results

### Treatment and Behavioral Studies

**Figure 1:**
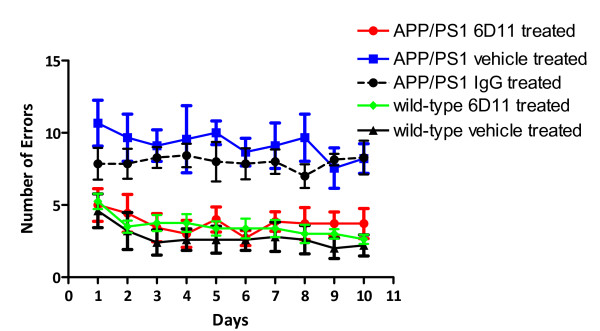
**Radial Arm Maze Cognitive Testing**. Figure 1 shows the results of radial arm maze cognitive testing. The number of errors is plotted versus the day of testing. Two-way ANOVA revealed a significant treatment effect in Tg 6D11 treated (n = 10) versus vehicle treated (n = 8) or murine IgG treated (n = 9) APP/PS1 Tg mice (*p *< 0.0001) with a Bonferroni *post-hoc *analysis showing no difference between Tg 6D11 treated and wild-type mice which were injected with either PBS alone (n = 8) or 6D11 (n = 9). APP/PS1 Tg non-treated mice and APP/PS1 mice treated with mouse IgG made significantly more errors than wild-type animals and 6D11 treated Tg mice (*p *< 0.01).

Cognitive ability was assessed by the number of errors (entry to previously visited arms) in consuming all 8 rewards using the radial arm maze (Figure 1). Statistical analysis by two-way ANOVA revealed a significant treatment effect in Tg 6D11 treated versus vehicle treated mice (*p *< 0.0001) with a Bonferroni *post-hoc *analysis showing no difference between Tg 6D11 treated and wild-type mice which were injected with either PBS alone or 6D11. APP/PS1 Tg vehicle treated mice and APP/PS1 mice treated with mouse IgG made significantly more errors than wild-type animals and 6D11 treated Tg mice (*p *< 0.01). The APP/PS1 Tg groups given vehicle (phosphate buffered saline [PBS]) or mouse IgG did not show statistically significant differences.

### Immunohistochemical Analyses for Amyloid Burden by Stereology

**Figure 2:**
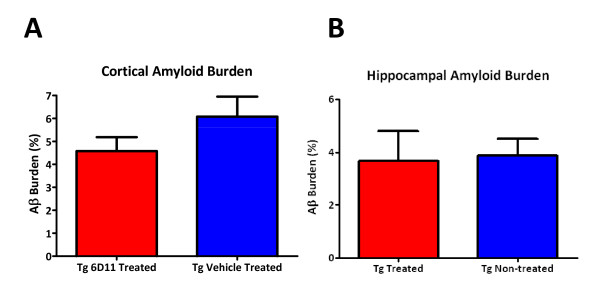
**Bar Graphs of Amyloid Quantitation by Stereology**. A and B shows a bar graphs of the amyloid quantitation by stereology in the cortex (A) and hippocampus (B) of Tg vehicle injected (n = 8) and 6D11 treated Tg mice (n = 10). There were no significant differences in the amyloid burden (% area occupied by 6E10 immunoreactivity) in both the cortex and hippocampus.

**Figure 3:**
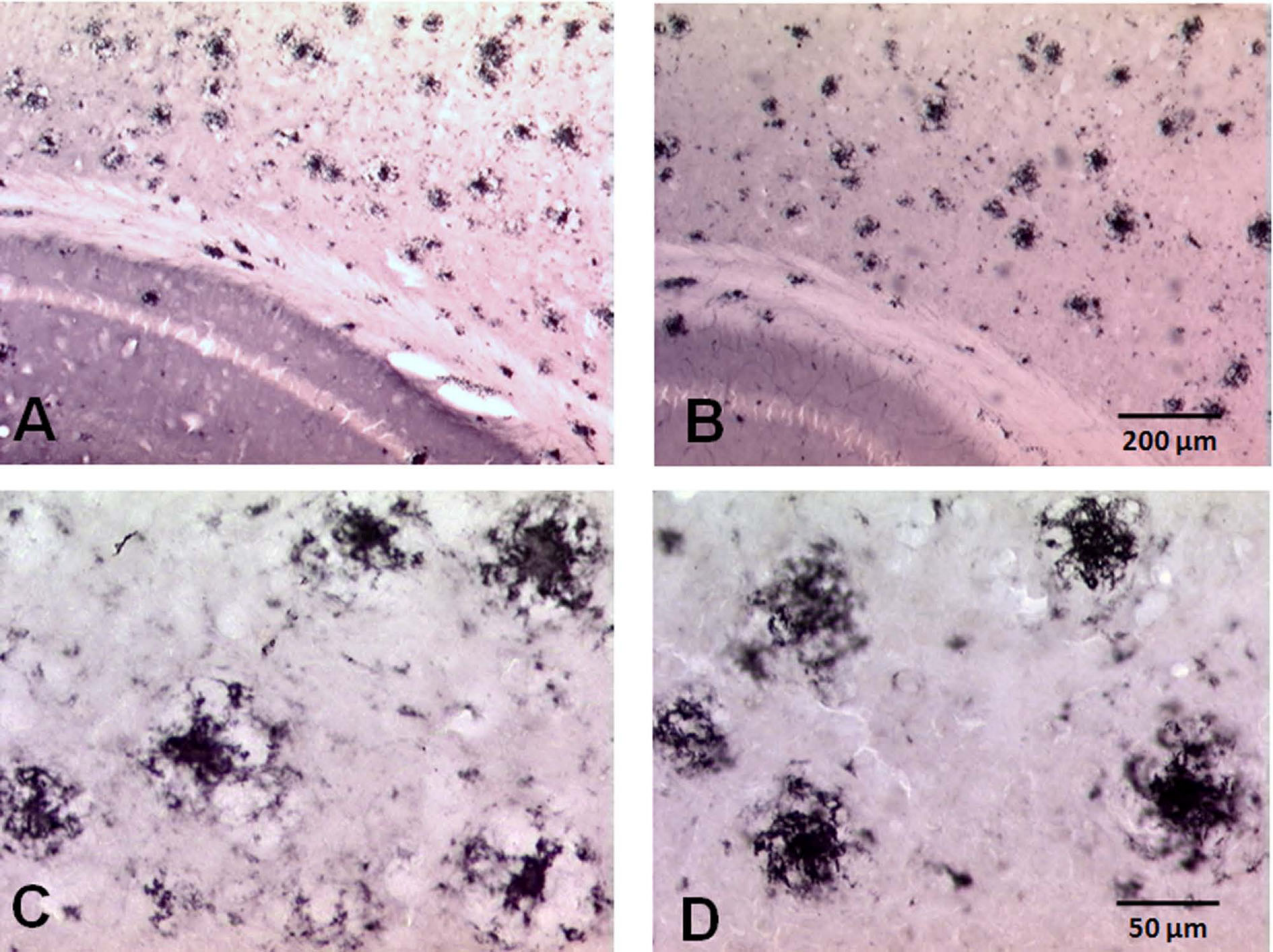
**Representative Sections Immunostained with anti-Aβ Antibody**. A-D show representative immunostained sections with anti-Aβ antibody 6E10 in the cortex and hippocampus at low power (A and B) and in the cortex (C and D) at higher magnification of 6D11 treated Tg mice (A and C) and vehicle injected Tg mice (B and D). Scale bar = 200 μm for A and B. Scale bar = 50 μm for C and D.

Immunohistochemistry of tissue sections revealed no significant difference in amyloid plaque burden in both the cortex and hippocampus of 6D11 treated Tg versus vehicle treated Tg mice using stereological methods (Figures 2 and 3).

### Immunohistochemical Analyses for Synaptic Density

**Figure 4:**
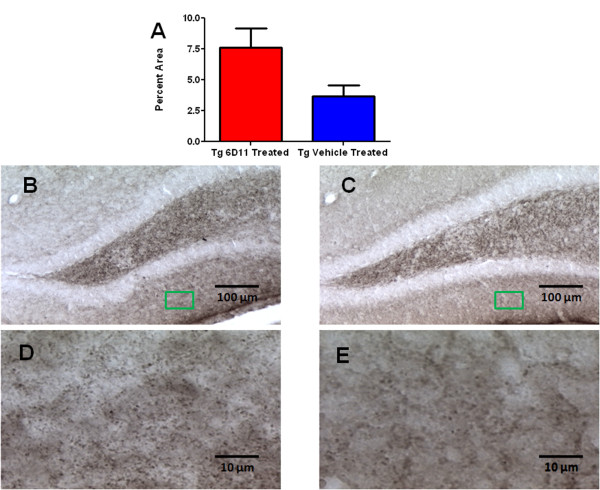
**Quantitation of Synaptophysin Immunoreactivity**. A shows a bar graph representation of synaptophysin immunoreactive presynaptic terminals in the molecular layer of the dentate gyrus of the hippocampus. The differences between 6D11 treated Tg mice (n = 10) and vehicle treated Tg mice (n = 8) are statistically significant by one-tailed *t*-test (*p *= 0.0267). B-E show representative sections immunostained with anti-synaptophysin antibody in the hippocampus at 10x magnification (B and C- Scale bar = 100 μm) and at 100x magnification (D and E- Scale bar = 10 μm) with the green box indicating the area of molecular layer magnified to the higher power. Images are of representative 6D11 treated Tg mice (B, D) and vehicle treated Tg mice (C, E).

Histological sections showed statistically significant greater synaptophysin immunoreactivity in the molecular layer of the dentate gyrus of the hippocampus among Tg mice treated with 6D11 monoclonal antibody versus vehicle treated Tg animals (*p *= 0.0267 by one-tailed *t*-test) (Figure 4).

### Tissue homogenization and sandwich ELISA for Aβ levels

**Figure 5:**
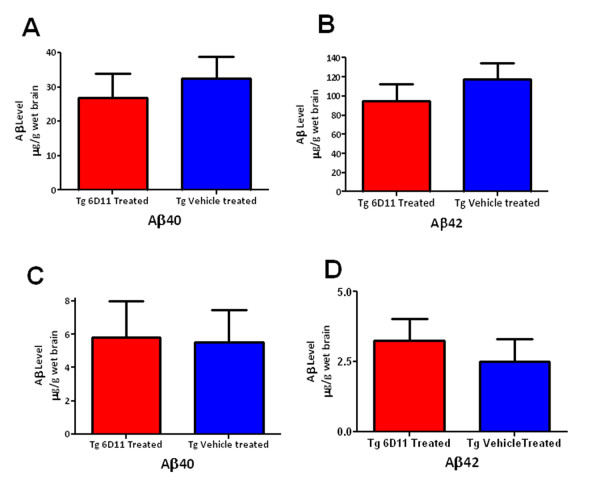
**Aβ40/42 Quantitation Biochemically**. Shows the levels of Aβ40 and Aβ42 in the FA and DEA extracted material from brains of vehicle treated Tg (n = 8) and 6D11 treated Tg mice (n = 10). Levels from the FA extract fraction of Aβ40 and Aβ42 are shown in A and B, respectively. Levels from the DEA extract fraction of Aβ40 and Aβ42 are shown in C and D, respectively. There were no significant differences in the levels of Aβ40 or Aβ42 in either the FA or DEA fractions.

ELISA results for 6D11 treated Tg versus vehicle treated Tg mice revealed no significant differences in Aβ levels for either formic acid (FA) treated (total Aβ fraction) or diethylamine (DEA) treated (soluble Aβ fraction) brain homogenates by two tailed *t*-test (Figure 5). Analysis of plaque-associated amyloid-β levels in FA treated homogenates presented similar levels in both Tg mouse groups for both Aβ40 and Aβ42. DEA treatment extraction of non-plaque associated soluble Aβ from prepared homogenates also showed comparable levels of soluble Aβ40 or Aβ42. There was a slight trend toward lowered Aβ40/42 levels in FA-treated brain homogenates, and raised Aβ40/42 levels in DEA-treated brain homogenates for 6D11 treated Tg animals; however, the differences were not statistically significant.

### Western blot detection and quantification of Aβ oligomers and aggregated Aβ

**Figure 6:**
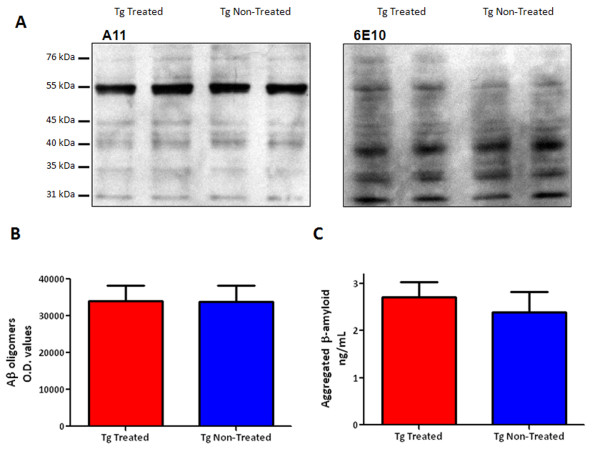
**Quantitation of Aβ Oligomer and Aggregated Aβ Levels**. A shows a Western blot using Aβ oligomer specific antibody A11 on the left from each of two representative 6D11 treated and vehicle treated Tg mice. On the right, a Western blot using anti-Aβ monoclonal antibody 6E10 is shown. B shows a bar graph of the densitometric analysis of the major A11 immunoreactive band at ~55 kDa, in arbitrary O.D. units. C shows a bar graph of the levels of aggregated Aβ in 6D11 treated (n = 10) and vehicle treated Tg mice (n = 8) as determined by ELISA. There is no significant difference between the 6D11 treated and vehicle treated Tg mice in the levels of Aβ oligomers determined by Western blot or of aggregated Aβ determined by ELISA.

Levels of Aβ oligomers in brain homogenates of 6D11 treated Tg versus vehicle treated Tg mice were detected by oligomer-specific polyclonal antibody, A11 [11](Figure 6A. left), and then subjected to densitometric analysis. Semiquantitation of A11 immunoreactive oligomers (~55 kDa) shows no significant difference between the groups (Figure 6B). The specificity of A11 blotting was confirmed by stripping the membrane and probing with anti-Aβ 6E10 monoclonal antibody [12](Figure 6A, right). There were no significant differences in the levels of aggregated Aβ peptides in 6D11 treated versus vehicle treated Tg mice as determined by ELISA (Figure 6C).

### Western Blot Detection and Quantification of PrP^C^

**Figure 7:**
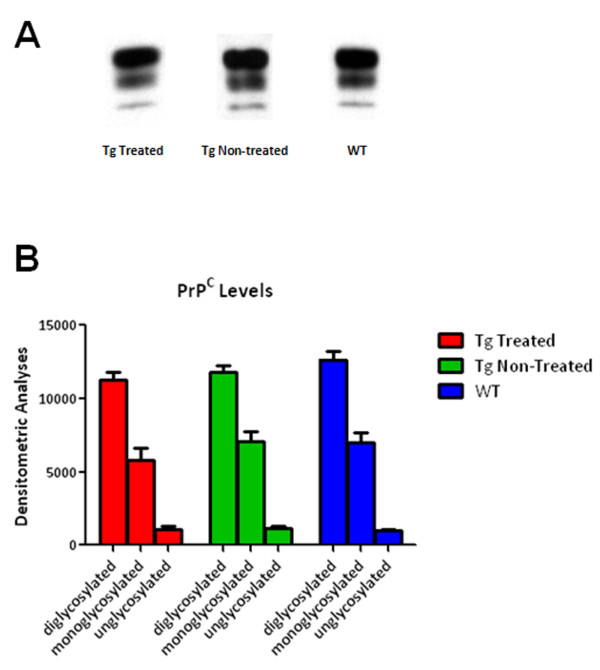
**Quantitation of PrP^C ^Levels**. A shows representative Western blots using anti-PrP mAb 6D11 showing immunostainging bands corresponding to the non-, mono-, and diglycosylated isoforms of PrP^C ^from a Tg 6D11 treated Tg mouse, vehicle treated Tg mouse and a wild-type mouse. B depicts a bar graph of the densitometric analysis (in arbitrary units) of the non-, mono-, and diglycosylated PrP^C ^bands. There are no significant differences the mouse groups in the levels of PrP^C^.

Semiquantitative analysis for areas under the curves representing di-, mono-, and non-glycosylated bands of PrP^C ^were similar among all three groups (Figure 7). Two-way ANOVA analysis showed no significant differences between 6D11 treated Tg, vehicle treated Tg and wild type control mice for all isoforms of PrP^C^.

## Discussion

We demonstrate that short term administration of anti-PrP mAb 6D11 is able to reverse cognitive deficits in an AD Tg mouse model, as determined by radial arm maze testing. Previous studies have shown that Aβ oligomers made from synthetic Aβ peptides bind to PrP^C ^and suppression of LTP in mouse hippocampal slice cultures could be abrogated by mAb 6D11, due to blocking the binding of oligomers to PrP^C ^[6]. In addition a recent study has shown that expression of PrP^C ^is required for the manifestation of cognitive deficits in an APP/PS1 Tg mouse model of AD, as determined by Morris water maze testing [8]. In this study APP/PS1 Tg mice were crossed onto a PrP^C ^knock-out (KO) background and it was found that these mice behaved similarly to wild-type mice despite having equivalent Aβ and amyloid β precursor protein (APP) levels to APP/PS1 Tg mice expressing PrP^C ^[8]. In the current study we show that just two weeks of treatment with 6D11 *in vivo *can have major cognitive benefits. It is important that this effect occurs without any significant change in the amyloid burden or Aβ peptide levels, determined by stereological and biochemical methods. This is not surprising since past studies of amyloid directed therapeutic interventions, such as vaccination, have shown in AD Tg mouse models that behavioural benefits often do not correlate with the overall amyloid burden but with Aβ oligomer levels [13-16]. In this study, we also have not altered Aβ oligomer or aggregated Aβ levels. The likely mechanism of action of the behavioural improvement in the 6D11 treated Tg mice is by blocking the binding of Aβ oligomers to PrP^C^. This is consistent with a critical role of PrP^C ^for mediating Aβ oligomer toxicity. Importantly we show using unbiased stereology that the 6D11 treatment in the APP/PS1 Tg mice was associated with greater synaptophysin immunoreactivity in the hippocampus compared to vehicle treated Tg mice. Hence 6D11 treatment ameliorated loss of synaptophsin immunoreactivity. Synaptic loss is a hallmark of AD which correlates best with the cognitive status of patients, as demonstrated in many studies using immunoreactivity of the presynaptic marker, synaptophysin [17,18]. Reduced synaptophysin immunoreactivity has also been detected in APP/PS1 Tg mouse models, which can be prevented by Aβ plaque and Aβ oligomer reducing interventions such as immunotherapy [19,20]. It is likely that the behavioural rescue in the 6D11 treated APP/PS1 mice is related to a greater synaptic density compared to Tg controls, as quantitated by synaptophysin immunoreactivity.

It has been suggested that PrP^C ^may be capable of binding other oligomeric species and function physiologically as a general "aggregation receptor" [21]. If this is true, application of mAbs such as 6D11 or other compounds which block oligomer binding to PrP^C ^at the 6D11 epitope (residues 93 to 109 [22]) could have therapeutic effects for a range of neurodegenerative conformational disorders. Interestingly, we have recently shown that 6D11 is therapeutically active *in vitro *and *in vivo *for prion disease using tissue culture and mouse models of prion infection [23,24]. Prion infection is dependent on binding between PrP^C ^and PrP^Sc^, with transmission of the abnormal conformation to the normal PrP^C^. The 6D11 epitope is important for this interaction [23], with PrP^Sc ^having an abnormal conformation with high β-sheet content similar to Aβ oligomers. Monoclonal antibodies with epitopes to all the different regions of PrP^C ^have been screened for inhibition of prion infection [25,26]. Of the few anti-PrP mAbs with therapeutic activity, several of these have an epitope at or near the 6D11 epitope [25], highlighting the importance of this region of PrP^C ^for binding to protein structures with an abnormal β-sheet conformation.

A recent study suggested that PrP^C ^is not essential for Aβ oligomer related toxicity, while confirming the high affinity binding between Aβ oligomers and PrP^C ^[9]. This study used very different methods from what is reported here. In the latter study mice which did not have any AD related pathology from human transgene expression were injected directly into brain ventricles using Aβ oligomers derived from synthetic Aβ peptides. This represents a sub-optimal animal model for AD pathology [20,27]. Furthermore, what represents a biologically relevant Aβ oligomer preparation is a subject of some debate with Aβ peptide assemblies ranging in mass from dimers up to multimers of ~1 MDa having been reported as neurotoxins using *in vitro *assays [28-32]. In our study we demonstrate blocking of cognitive deficits related to *in vivo *generated Aβ oligomers. The Balducci et al. studies also used novel object recognition testing in contrast to radial arm maze or water maze spatial memory testing [9]. Most behavioural studies using AD Tg models examine spatial memory with radial or water maze testing [20,27], with some studies in AD Tg mice having shown impairments of spatial memory but not of object recognition [33]. In our own past studies of behaviour where we have used novel object recognition in AD Tg mice, this has been a less sensitive measure that is more open to confounding variables [16,34]. Hence, these significant methodological differences likely explain the contrasting results. In addition, the recent report that crossing an APP/PS1 Tg mouse onto a PrP^C ^KO rescues the mice from any cognitive deficit, despite there being no change to the Aβ or amyloid precursor protein levels, clearly points to the importance of PrP^C ^in mediating Aβ related toxicity [8]. Our findings are consistent with this report, which also showed that the synaptic density in the APP/PS1 Tg mice on a PrP KO background was greater compared to controls, using synaptophysin immunoreactivity. However, this is a controversial and complex area of research, since it is likely that Aβ oligomers mediate toxicity via multiple, non-mutually exclusive pathways and the results obtained depend on the experimental setting [35-37].

In our study we used very large doses of 6D11. This was because only a small fraction of peripherally injected mAb would be expected to cross the blood brain barrier (BBB). Prior studies have established that about ~0.1% of the injected dose of IgG anti-Aβ antibodies cross the BBB and that this small faction can have a significant biological effect [38-40]. We presume that a similar percentage of our 6D11 was also able to cross the BBB. We controlled for possible non-specific effects on behaviour of injecting such large doses of 6D11 or murine IgG by including a group of APP/PS1 mice which were injected with an equivalent dose of murine IgG and a group of wild-type mice injected with the same dose of 6D11. The APP/PS1 Tg mice given the murine IgG performed similarly on the radial arm maze to the Tg mice given vehicle, while the 6D11 injections had no demonstrable behavioural effect in the wild-type mice compared to wild-type mice given vehicle injections. Future development of single chain variable region (scFv) antibodies based on 6D11 or peptidomimetics which also block Aβ oligomer binding to PrP^C ^at the 6D11 epitope, may lead to agents which are even more effective and have better pharmacokinetic properties. However, it is striking that a relatively short term administration of 6D11 over a period of 2 weeks was able to reverse behavioural deficits associated with a high amyloid burden and high Aβ40/42 levels. This suggests the significant potential for blocking the PrP^C^-Aβ oligomer interaction as a therapeutic intervention in pre-existing AD. This is in contrast to approaches such as vaccination, which are in current clinical trials [41]. Immunomodulation has been shown in AD Tg mouse studies to be much less effective or ineffective with more advanced disease [16] and in more limited studies in AD patients amyloid removal has not been associated with any significant cognitive benefits [42,43]. The limited autopsy data from the initial human active vaccination trial targeting fibrillar Aβ plaque deposits showed that patients had partial or near complete plaque removal and a reduction of Aβ load compared to age matched non-immunized controls. However, there were no differences between placebo and active immunization groups in the long-term survival outcome, time to severe dementia and in cognitive outcome measurements such as ADAS-Cog, MMSE or DAD [42]. In living patients, part of a passive immunization trial targeting Aβ, a 25% amyloid reduction versus controls was documented using PET imaging methods, in the absence of measurable cognitive benefits [43]. These studies highlight the importance of developing interventions which directly target Aβ oligomers or downstream toxicity related to Aβ oligomer interactions, such as the approach described here.

## Conclusions

We demonstrate in an AD Tg mouse model that infusion of an anti-PrP^C ^mAb, produces a significant behavioural rescue in the setting of advanced disease, even with a relatively short treatment regiment. We presume the mechanism of action is by blocking the binding between Aβ oligomers and PrP^C^, resulting in an amelioration of synaptic loss. This finding opens a novel therapeutic approach for AD and perhaps for other conformational neurodegenerative disorders.

## Methods

### Treatment of APP/PS1 Tg Mice

APP/PS1 Tg mice [10] aged 8 months were either treated with anti-PrP monoclonal antibody 6D11 (n = 10) or given phosphate-buffered saline (PBS) (n = 8) or given mouse IgG (n = 9). These APP/PS1 Tg mice carry a Swedish K670L/M671L APP mutation and a presenilin 1 M146L mutation [10]. By 8 months of age these APP/PS1 Tg mice already have abundant Aβ deposition in the form of plaques [10]. 6D11 is an anti-PrP mouse monoclonal antibody (mAb) which recognizes residues 93 to 109 of mouse PrP^C ^with residues 97 to 100 being the primary determinant for binding [22]. This region of PrP^C ^is very homologous in human PrP^C^; hence, 6D11 also recognizes human PrP^C ^[23]. Wild-type mice were given PBS i.p. or the same dose of 6D11 as controls. Treatments consisted of 1 mg injections 5 times a week for 2 weeks of either the 6D11 or mouse IgG (Invitrogen, catalogue number 10400C). The mouse IgG was dialyzed against PBS prior to *in vivo *use in order to remove the sodium azide in which it was supplied. Tg 6D11 treated (n = 10), Tg mouse IgG treated (n = 9), Tg PBS injected (n = 8), wild-type 6D11 treated (n = 9) and wild-type PBS control mice (n = 8) were subjected to behavioural testing by radial arm maze to assess the effect of 6D11 treatment on cognition by spatial learning. The 6D11 or control injections continued during the behavioural analysis. All mouse care and experimental procedures were approved by the Institutional Animal Care and Use Committee at the New York University School of Medicine.

### Behavioural Analyses

Spatial learning (working memory) was evaluated using an eight-arm radial maze with a water well at the end of each arm, as we have previously reported [15,44,45]. Clear Plexiglas guillotine doors, operated by a remote pulley system, controlled access to the arms from a central area from which the animals entered and exited the apparatus. After 2 days of adaptation, water-restricted mice (2 h daily access to water) were given one training session per day for ten consecutive days. For each session, all arms were baited with 0.1% saccharine solution, and animals were permitted to enter all arms until the eight rewards had been consumed. The number of errors (entries to previously visited arms) and time to complete each session were recorded. The behavioral testing was performed by an individual blinded to the animal's treatment status.

### Immunohistochemical Analyses for Amyloid Burden

Mice were anesthetized with sodium pentobarbital (150 mg/kg i.p.) and perfused transaortically with heparinized phosphate buffered-saline, and the brains processed. The right hemisphere was immersion-fixed in periodate-lysine-paraformaldehyde, while the left hemisphere was snap frozen for measurements of Aβ40/42 peptide, PrP^C ^and Aβ oligomer levels. After fixation, brains were placed in 2% DMSO/20% glycerol in PBS and stored until sectioned. Serial coronal sections of 40 μm were cut and every fifth section stained with 6E10, a monoclonal antibody that recognizes Aβ and immunolabels both pre-amyloid and Aβ plaques as we have previously described [34,45]. Sections were incubated in 6E10 at a 1:1000 dilution and anti-synaptophysin SY38 at a 1:2000 dilution (Millipore, MA). A mouse-on-mouse immunodetection kit (Vector Laboratories, Burlingame, CA) was used with the anti-mouse IgG secondary antibody at a 1:3000 dilution. Antibody staining was revealed with 3,3'-diaminobenzidine tetrahydrochloride (DAB, Sigma-Aldrich) with nickel ammonium sulfate (Ni; Mallinckrodt, Paris, KY) intensification.

### Image Analyses for Amyloid Burden and Synaptophysin Immunoreactivity

Immunohistochemistry of tissue sections was quantified with a Bioquant image analysis system (BIOQUANT Image Analysis Corporation, Nashville, TN), and unbiased sampling was used, as previously published [44,45]. Seven sections were analyzed per animal. All procedures were performed by an individual blinded to the experimental condition of the study. Total A[001] burden (defined as the percentage of test area occupied by Aβ) was quantified for the cortex and for the hippocampus on coronal plane sections stained with the monoclonal antibody 6E10. Intensification with nickel ammonium sulfate resulted in black Aβ with minimal background staining that facilitated threshold detection. The cortical area was dorsomedial from the cingulate cortex and extended ventrolaterally to the rhinal fissure within the right hemisphere. Test areas (640[001] μm × 480 μm) were randomly selected by applying a grid (800 μm × 800 μm) over the traced contour. Hippocampal measurements (600 μm × 600 μm) were performed similarly to the cortical analysis [34,44]. Synaptophysin positive presynaptic terminals were counted bilaterally in the molecular layer of the dentate gyrus of the hippocampus using a 100× objective of at least three hippocampal sections in each mouse comparing the 6D11 treated and PBS injected APP/PS1 Tg mice. The fractional area occupied by the immunoreactive puncta was measured as previously described using a Bioquant image analysis system [8,46].

### Tissue homogenization and sandwich ELISA for Aβ levels

Brain homogenates, 10% (w/v), were prepared in 20 mmol/L Tris, pH 7.4, 250 mmol/L sucrose, 1 mmol/L EDTA, and 1 mmol/L EGTA. Immediately before use, 1:100 volume of 100 mmol/L PMSF solution (in ethanol) and 1:1000 volume of LAP (5 mg each of leupeptin, antipain, and pepstatin A per milliliter of *N-N*- dimethylformamide) was added to the homogenization buffer, as we have previously described [44,45]. For extraction of soluble Aβ, brain homogenates were thoroughly mixed with an equal volume of 0.4% diethylamine/100 mmol/L NaCl and centrifugation at 135,000 × *g *for 1 hr at 4 °C and subsequently neutralized with 1:10 volume of 0.5 mol/L Tris, pH 6.8, followed by aliquoting, flash-freezing on dry ice, and storage at -80°C until analysis. Samples were also treated with formic acid (95%, Sigma) for extraction of total Aβ. Homogenates (200 μl) were added to 440 μl cold formic acid (FA) and sonicated for one minute on ice. Subsequently, 400 μl of this solution was spun at 100,000 × g for 1 hour at 4 °C. Then, 210 μl of the resulting supernatant was diluted into 4 ml of FA neutralization solution (1 M Tris base, 0.5 M Na_2_HPO_4_, 0.05% NaN_3_), aliquoted, flash-frozen on dry ice and stored at -80°C until used for Aβ measurements.

The total and soluble Aβ levels were measured using a combination of mouse monoclonal antibody 6E10 (specific to an epitope present on amino acid residues 1 to 16 of Aβ) and two different rabbit polyclonal antibodies specific for either Aβ40 (R162) or Aβ42 (R165), in a double-antibody sandwich ELISA as described previously [44,45]. The optical density (OD) was measured at 450 nm. The relationship between OD and Aβ peptide concentration was determined by a four-parameter logistic log function. Non-linear curve fitting was performed with the KinetiCalc program (Biotek Instruments, Inc., Winooski, VT) to convert OD of plasma to estimated concentrations. The assay was performed by an investigator (PM) blinded to group assignment. The levels of Aβ species are presented as μg of Aβ per g of wet brain, taking into account dilution factors introduced by multiple steps throughout the assay (brain homogenization and extraction procedures).

### Western Blot Detection and Quantification of Aβ Oligomers

Samples of brain homogenate were centrifuged at 100,000 × *g *for 1 hour, and the total protein concentration in the supernatant was estimated by using the Bicinchoninic acid assay (BCA; Pierce, Rockford, IL), as we have previously described [44,45]. Samples (40 μg of total protein), mixed with an equal volume of Tricine sample buffer, were electrophoresed on 12.5% Tris-tricine polyacrylamide gels (under nonreducing conditions) and transferred to nitrocellulose membranes. The blots were blocked with 5% nonfat dry milk in Tris-buffered saline Tween 20 (TBS-T) for 2 hours at room temperature. Oligomer-specific A11 polyclonal antibody (Biosource, Camarillo, CA) was diluted (1:1000) in 0.1%BSA/TBS-T and incubated with the blots for 2 h at room temperature. Bound antibody was visualized with horseradish peroxidase-conjugated goat anti-rabbit IgG (1:8000; 1 h, Pierce, Rockford, IL) and the ECL detection system (Pierce, Rockford, IL). The specificity of A11 staining was confirmed by probing the membrane with anti-Aβ monoclonal antibodies 6E10 or 4G8 [44]. Densitometric analysis of A11 immunoreactive oligomer specific bands was performed with NIH Image J version 1.34 software.

### Western Blot Detection and Quantification of PrP^C^

Brain samples were weighed, homogenized and sonicated (10% w/v) in a buffer containing 20 mM Tris pH 7.5, 250 mM sucrose, 1 mM EDTA, 1 mM EGTA, and Complete^® ^protease inhibitor (Boehringer-Mannheim, Indianapolis IN). Samples were centrifuged for 3 min at 10,000 × g at 4°C to remove cellular debris. The total protein concentration was assayed by the BCA method as described above. If not used immediately, supernatants were divided into 100 μl aliquots, which were flash frozen and stored at -80°C. Semi-quantitative Western-blot was used to compare the relative content of PrP^C ^among samples containing matched amounts of total protein. Aliquots of brain homogenates containing 20 μg of the total proteins were titrated by adding sample buffer to a final protein concentration of 1 μg/1 μl. Samples were subjected to SDS-PAGE and Western-blotting into nitrocellulose membranes where PrP^C ^was detected with Mab 6D11 (0.05 μg/ml) as described previously [23]. For the densitometric analysis, the exposure time of Western blot membranes was kept standard in all experiments at 30 seconds. Developed films were converted into 8 bit grayscale digital files using a Epson Perfection 4990 scanner (Epson America; Long Beach, CA) and Adobe Photoshop software 7.01 (Adobe Systems; San Jose, CA) and saved in a TIF format with a resolution of 600 dpi. Quantification of PrP^C ^was performed using NIH Image J software v 1.34. Areas under the curves for three PrP^C ^bands representing non-, mono-, and diglycosylated isoforms of the protein were analyzed from each sample.

### Sandwich ELISA for Aggregated Aβ

Aggregated Aβ levels were determined using an Invitrogen Aggregated Aβ kit which uses a solid phase sandwich ELISA (Invitrogen, Camarillo, CA). This was done following the manufacturer's instructions. In brief, a monoclonal antibody specific for the N-terminus of human Aβ was pre-coated onto wells of the provided microtiter strips. Samples diluted in the provided standard diluent buffer were measured against a standard containing aggregated Aβ. Samples were incubated for 2 hrs at room temperature allowing the Aβ to bind the capture antibody, followed by extensive washing. Incubation with biotinylated detector antibody (same monoclonal antibody coated onto wells) for 1 hr at RT served as a detection antibody by binding to the immobilized aggregated Aβ. After removal of excess antibody, horseradish peroxidase-labelled streptavidin (SAV-HRP) was allowed to incubate for 30 min, followed by washing, after which tetramethylbenzidine (TMB) substrate was added to produce a colorimetric solution. The TMB reaction was stopped and the absorbance of each well was read at 450 nm. The standards provided a linear curve and the best-fit line determined by linear regression was used to calculate the concentration of aggregated Aβ in samples.

### Data Analysis

The amyloid burden, the levels of Aβ40/42 peptides within the brain, Aβ oligomers and aggregated Aβ levels were analyzed by unpaired two-tailed Student's *t*-tests (GraphPad Prism, version 5; GraphPad Inc., San Diego, CA, USA). The synaptophysin immunoreactivity was compared by one-tailed Student's *t*-test (GraphPad Prism). The radial arm maze data was analyzed by two-way ANOVA repeated measures and a Bonferroni *post hoc *test (GraphPad Prism). The PrP^C ^band densitometry was also analyzed by two-way ANOVA (GraphPad Prism).

## Authors' contributions

EC performed the mouse injections, the histology, the biochemical extractions, the image analysis and the Aβ oligomer measurements. YJ performed the behavioral studies. YS performed the mouse breeding and genotyping. RJK and RBK provided the purified 6D11 antibody. PDM performed the Aβ40/42 ELISA measurements. SMS provided critical review of the manuscript and made the original observation of Aβ oligomer binding to PrP^C^. TW planned and designed the experiment and wrote the manuscript. All authors read and approved the final manuscript.
